# Advancements in the Development of HIF-1α-Activated Protein Switches for Use in Enzyme Prodrug Therapy

**DOI:** 10.1371/journal.pone.0114032

**Published:** 2014-11-26

**Authors:** R. Clay Wright, Arjun Khakhar, James R. Eshleman, Marc Ostermeier

**Affiliations:** 1 Department of Chemical and Biomolecular Engineering, Johns Hopkins University, Baltimore, Maryland, United States of America; 2 Department of Biomedical Engineering, Johns Hopkins University, Baltimore, Maryland, United States of America; 3 Departments of Pathology and Oncology, Sol Goldman Pancreatic Cancer Center, Johns Hopkins University School of Medicine, Baltimore, Maryland, United States of America; Columbia University, United States of America

## Abstract

While gene-directed enzyme prodrug therapy has shown potential as a cancer therapeutic in animal and clinical trials, concerns over the efficacy, selectivity, and safety of gene delivery vehicles have restricted its advance. In an attempt to relieve some of the demands on targeted gene delivery vehicles and achieve the full potential of enzyme prodrug therapy, cancer-targeted activity can be engineered into the enzyme itself. We previously engineered a switchable prodrug-activating enzyme that selectively kills human cancer cells accumulating the cancer marker hypoxia-inducible factor-1α (HIF-1α). This HIF-1α-activated protein switch (Haps59) is designed to increase its ability to convert the prodrug 5-fluorocytosine into the chemotherapeutic 5-fluorouracil in a HIF-1α-dependent manner. However, in cancer cell lines expressing Haps59 the 5FC sensitivity difference between the presence and absence of HIF-1α was not as large as desired. In this work, we aimed to improve the cancer specificity of this switch via a directed evolution approach utilizing random mutagenesis, linker mutagenesis, and random insertion and circular permutation. We identified improved HIF-1α-activated protein switches that confer *E. coli* with modest increases in HIF-1α-dependent 5FC toxicity. Additionally, the current bottleneck in the development of improved HIF-1α-activated protein switches is screening switch candidates in mammalian cells. To accommodate higher throughput and reduce experimental variability, we explored the use of Flp recombinase-mediated isogenic integration in 293 cells. These experiments raised the possibility that Haps59 can be activated by other interactors of the CH1 domain, and experiments in *E. coli* indicated that CITED2 can also activate Haps59. Although many CH1 binding partners are also oncogenes, CH1's promiscuous binding and subsequent off-target activation of Haps59 needs to be examined under normal physiological conditions to identify off-target activators. With aberrant activating molecules identified, further directed evolution can be performed to improve the cancer specificity of HIF-1α-activated protein switches.

## Introduction

Despite the expansive development of targeted cancer therapies, traditional chemotherapeutics are still used during the course of treatment against most cancers. Some chemotherapeutics, such as 5-fluorouracil, methotrexate, and taxol exert selective toxicity by exploiting the increased metabolism of cancer cells. However, the therapeutic window—the dosage range that can effectively kill cancer cells while leaving normal cells intact—is very narrow for chemotherapeutics. Even within this window side effects associated with toxicity to the digestive system, immune system, and hair follicles can be devastating to patients. Still, traditional chemotherapy remains one of the few ways to effectively treat patients with metastatic cancer [1].

Enzyme prodrug therapy (EPT) attempts to limit off-target toxicity by producing the active form of a chemotherapeutic selectively within cancer cells or the cancer microenvironment. By initially converting the chemotherapeutic into a non-toxic prodrug form, delivering a prodrug-activating enzyme selectively to cancer cells, and administering the prodrug systemically, the prodrug will be converted to its toxic chemotherapeutic form only within enzyme-containing cancer cells or their vicinity [2]. The prodrug-activating enzyme may be delivered in its protein or gene form, via direct injection or using cancer-selective antibodies [3], nanoparticles [4], stem cells [5], bacteria [6], or viruses [7]. An additional layer of tumor selectivity can be achieved via a tumor specific promoter, if the gene encoding the enzyme is delivered [8].

EPT has shown some success in animal studies [9],[10],[11], and has been demonstrated as safe in a handful of Phase I clinical trials [12],[13],[14],[15],[16]. However, all of the above studies either deliver the enzyme-encoding vector via direct intratumoral injection or prepare xenografts using cells previously transduced with the prodrug-activating enzyme. Systemic delivery of both the enzyme-encoding vector and prodrug may provide more comprehensive therapy by affecting distal metastatic sites as well as the primary tumor, while still minimizing negative side effects. This approach could replace more conventional chemotherapy. However, inefficiency and/or non-specificity of current targeted delivery methods limit this approach [17],[18].

**Figure 1:**
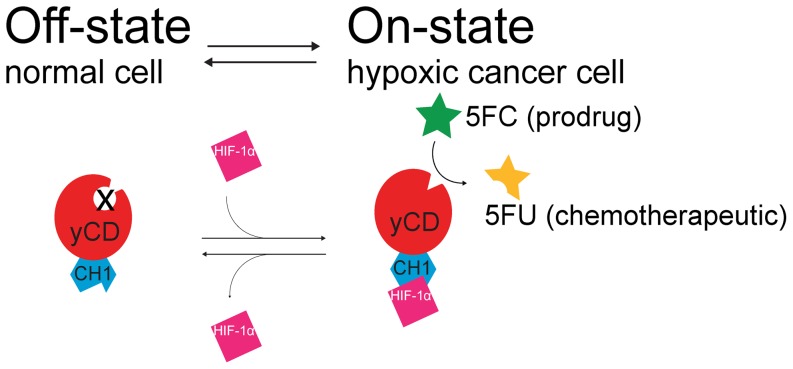
Schematic depiction of HIF-1α-Activated Protein Switches (Haps). These protein switches are composed of the prodrug-converting enzyme yeast cytosine deaminase (yCD), which converts the non-toxic prodrug 5-fluorocytosine (5FC) to the highly toxic chemotherapeutic 5-fluorouracil (5FU), and the HIF-1α-binding CH1 domain of the human p300 protein. The cancer marker, HIF-1α, modulates the enzymatic activity of the yCD-domain of the switch, either through an allosteric mechanism or through stabilizing the protein such that it accumulates at higher levels in the cell. In normal cells HIF-1α is absent and the yCD domain is inactive. In the presence of HIF-1α – ideally only in cancer cells – the yCD domain is active and able to produce the chemotherapeutic.

To subvert these limitations and provide an additional layer of cancer specificity we have previously demonstrated the use of protein switches in which the prodrug-activating enzymatic activity is switched on by a cancer marker [19]. The addition of targeting at the level of the therapeutic enzyme itself would allow use of the most efficient and systemic delivery systems enabling specific, comprehensive treatment. In previous work, we created a cancer-marker-activated enzyme by randomly inserting the coding sequence for a cancer-marker-binding domain into the sequence encoding a prodrug-converting enzyme and then selecting for those sequences encoding fusion proteins with cancer-marker-regulated enzymatic activity. We chose as the prodrug-converting enzyme a thermostable variant of yeast cytosine deaminase (yCD) [20]. yCD converts the non-toxic prodrug, 5-fluorocytosine (5FC), to the highly toxic chemotherapeutic, 5-fluorouracil (5FU), and similarly converts cytosine to uracil (Fig 1). This enzyme-prodrug pair has been shown to be very effective, even when the enzyme is delivered at low levels due to a strong bystander effect (diffusion of the activated chemotherapeutic from enzyme-containing cells to neighboring cells) [11],[21].

As the activating cancer marker we chose HIF-1α, due to its tight regulation bestowing nearly complete absence in normal tissue and its ubiquitous nature in cancer cells [22]. HIF-1α is necessary for tumors to recruit vasculature allowing them to grow larger than 2 mm in diameter [23]. Additionally HIF-1α is frequently overexpressed in metastatic cells [24] and is strongly associated with invasiveness [25]. As the cancer-marker-binding domain we chose the CH1 domain of the human p300 protein (Fig 1), a transcriptional coactivator involved in several signaling pathways associated with neoplastic progression. The CH1 domain binds HIF-1α with high affinity [26], as well as several other potential neoplastic biomarkers including p53 [27], CITED2 [28], and NF-κB/p65 subunit [29].

To select for cancer-marker-activated enzymes, we coexpressed the library of yCD-CH1 fusions along with the C-terminal activation domain (CTAD) of HIF-1α, which has been shown to interact strongly with the CH1 domain in *E. coli* [30],[31]. This library was subjected to two tiers of selection: a positive selection for cytosine deaminase activity in the presence of co-expressed HIF-1α-CTAD and a negative selection for the absence of 5FC deaminase activity in the absence of HIF-1α. Both selections were performed in GIA39 *E. coli* cells, which are devoid of native *E. coli* cytosine deaminase activity. Negative selections were performed on minimal media supplemented with 5FC and uracil (i.e. an active fusion in the absence of HIF-1α will convert 5FC to 5FU, which is toxic to bacteria, but cells with an inactive switch will be able to grow utilizing the supplemented uracil). Positive selections were performed in cells expressing the CTAD of HIF-1α on minimal media lacking uracil and supplemented with cytosine, forcing cells to rely on the cytosine deaminase of active fusions to generate uracil.

**Figure 2:**
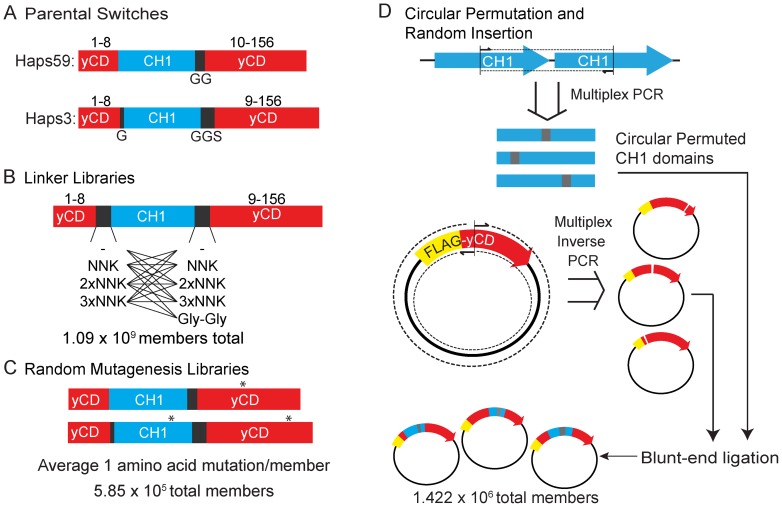
Strategies for creating improved HIF-1α-activate protein switches. (A) Sequences of parental switches Haps3 and Haps59. Numbers above indicate the amino acid residues in yCD. Letters below indicate the 1-letter abbreviation amino acid sequence of the corresponding linker. (B) Schematic of the linker libraries created in Haps59. Lines indicate which combinations of N- and C-terminal linkers were combined to comprise the 18 libraries. (C) Schematic of random mutagenesis libraries of Haps59 and Haps3. A * indicates a representative point mutation. (D) Schematic of the construction of libraries of random circular permutations of CH1 that are randomly inserted into the yCD domain of FLAG-tagged yCD. Multiplex PCR was used to create all possible circular permutations of the CH1 domain. Multiplex inverse PCR was used to create all possible insertion sites within yCD. These two populations of DNA were ligated to create the library.

Using these selections, we isolated two HIF-1α-activated protein switches (Haps) with very similar sequences, Haps3 and Haps59 (Fig 2A) [19]. In both switches the CH1 domain is inserted after the eighth residue of yCD. Compared to Haps59, Haps3 has slightly longer, more flexible linkers connecting the CH1 domain to the N- and C-terminal fragments of yCD. Despite their similarity, Haps3 and Haps59 possess different efficacies in both *E. coli* and cancer cell lines. Haps3 was consistently much more active than Haps59, in both the on-state (presence of HIF-1α) and off-state (absence of HIF-1α), resulting in Hap59 having a larger difference in on- and off-state activity than Haps3 did. A large difference between on- and off-states in *E. coli* is desirable because this should theoretically result in a larger therapeutic window. In this study, we sought to create enhanced ΗIF-1α-activated protein switches (Ehaps) via directed evolution, with the goal of expanding the therapeutic window of these switches and improving the chance of success in future translational studies.

## Results and Discussion

**Figure 3:**
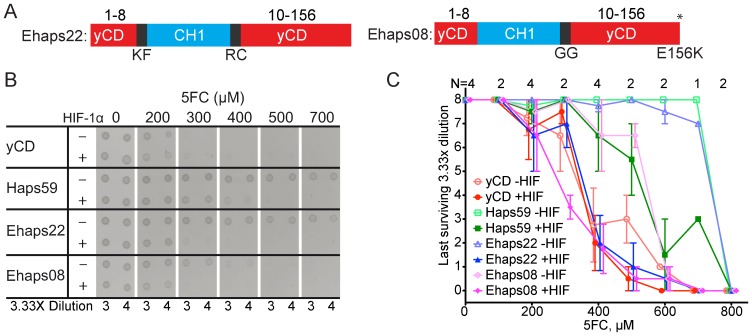
Characterization of Ehaps22 and Ehaps08 in *E. coli.* (A) Diagram of Ehaps22 and Ehaps08 sequences. Ehaps22 was selected from the linker library containing 2-amino acid N- and C-terminal linkers. Ehaps08 was selected from the random mutagenesis library of Haps59. Numbers above indicate the amino acid residues in yCD. Letters below indicate the 1-letter abbreviation of the amino acid sequence of the corresponding linker or mutation. (B) Representative 5FC dot toxicity assay on cells expressing yCD, Haps59, Ehaps22, or Ehaps08. Serial dilutions of equal density log phase cultures containing either pGA (−HIF-1α, i.e. not expressing HIF-1α) or pGA-HIF (+ HIF-1α, i.e. expressing HIF-1α) were spotted on minimal media plates containing increasing concentrations of 5FC. (C) Quantification of dot toxicity assay. The highest dilution at which growth was observed for each culture on each plate is plotted against the concentration of 5FC in each plate. The number of replicates of each plate is listed above. Error bars represent max and min for N = 2 plates and standard deviation for N = 4. See Fig S1, S2, S3, and S4 for replicate plates.

We created three types of libraries in an attempt to identify switches with properties superior to Haps59 via positive and negative genetic selections. These three libraries were 1) linker libraries in which the linker regions between the CH1 and yCD domains of Haps59 were varied in length and amino acid sequence (Fig 2B), 2) a random mutagenesis library of Haps59 (Fig 2C), and 3) a library designed to consist of all possible circular permutations of the CH1 domain inserted at every position in yCD (Fig 2D).

### Linker libraries yield minor improvements in HIF-1a-specific activity

Haps3 and Hap59 differ only in the linker regions joining the CH1 and yCD domains. Based on their different properties despite this similarity, we hypothesized that the length and composition of inter-domain linkers is crucial for optimizing switching activity. We created a total of 18 libraries designed to encompass all possible 1.14 billion unique combinations of N- and C-terminal linkers of 0, 1, 2, and 3 random amino acids as well as the wild-type Haps59 C-terminal Gly-Gly linker (Fig 2B) [32]. A total of 16.6 million members of these libraries were subjected to two-tiered selections under conditions designed to enrich for members with increased on-state activity and decreased off-state activity. The stringency of the positive selection was increased by a decrease in the amount of cytosine in the media. The stringency of the negative selection was increased by an increase in the concentration of 5FC. The majority of variants surviving the two-tiered selection had similar switching properties to Haps59 as assessed in *E. coli* cells. These variants predominantly had no N-terminal linker and a 2 amino acid C-terminal linker composed of bulky, hydrophilic amino acids (Table 1).

**Table 1:** List of linker library members with switching abilities similar to Haps59.

Clone	N-terminal Linker	C-terminal linker
Ehaps03	-	Arg-Arg
Ehaps05	-	Cys-Lys
Ehaps06	-	Gly-Val
Ehaps07	-	Ser-Arg
Ehaps12	-	Gly-Gln
Ehaps13	-	Gly-Pro
Ehaps16	-	Lys-Lys
Ehaps18	-	Gly-Arg
Ehaps31	Cys	Gly-Gly-Arg
Ehaps21	Ala-Val	Gly-Arg
Ehaps22	Lys-Phe	Arg-Cys
Ehaps32	Gly-Ser-Arg	Ala-Leu

After subcloning, all identified switches possessed essentially equivalent activity to Haps59 in *E. coli* except Ehaps22, which showed improved switch properties (Fig 3). Ehaps22 contains an N-terminal Lys-Phe linker and a C-terminal Arg-Cys linker (Fig 3A). The improved switching window of Ehaps22 results from a significant increase in the toxicity of 5FC in the presence of HIF-1α, up to a level approaching the toxicity of yCD (Fig 3B&C). The toxicity of 5FC in the absence of HIF-1α also increased but marginally.

Through linker mutagenesis we have identified several switches, one of which had an improved difference between its on- and off-states. We had hoped to identify switches in which the on-state activity was increased and the off-state activity was decreased. No isolates from the linker libraries exhibited both an improved off state and wider difference between the on- and off-states. However, increasing the overall activity of these switches does lessen the efficiency burden on delivery vehicles.

### Random mutagenesis increases overall activity without improving specificity

We hypothesized that mutations in Haps59 might be able to accentuate its switching properties. An error-prone PCR library of the *Haps59* gene was constructed under conditions designed to achieve on average a single non-synonymous mutation per variant. A total of 1.2 million members – representing in theory all of the single mutants and 19% of possible double mutants [32] – was subjected to selections at the same stringency levels as in the linker libraries. No variant with an improved switching window was identified. However, Ehaps08, a variant of Haps59 with an Asp to Lys mutation in the C-terminal residue (Fig 3A), exhibited increased activity in both the on- and off-states, while retaining a moderate therapeutic window (Fig 3B&C). Four separate colonies were isolated that contained this mutation, which resulted from a single G to A transition.

### Addition of a FLAG-tag abolishes activity in *E. coli* and hinders the creation of switches

**Figure 4:**
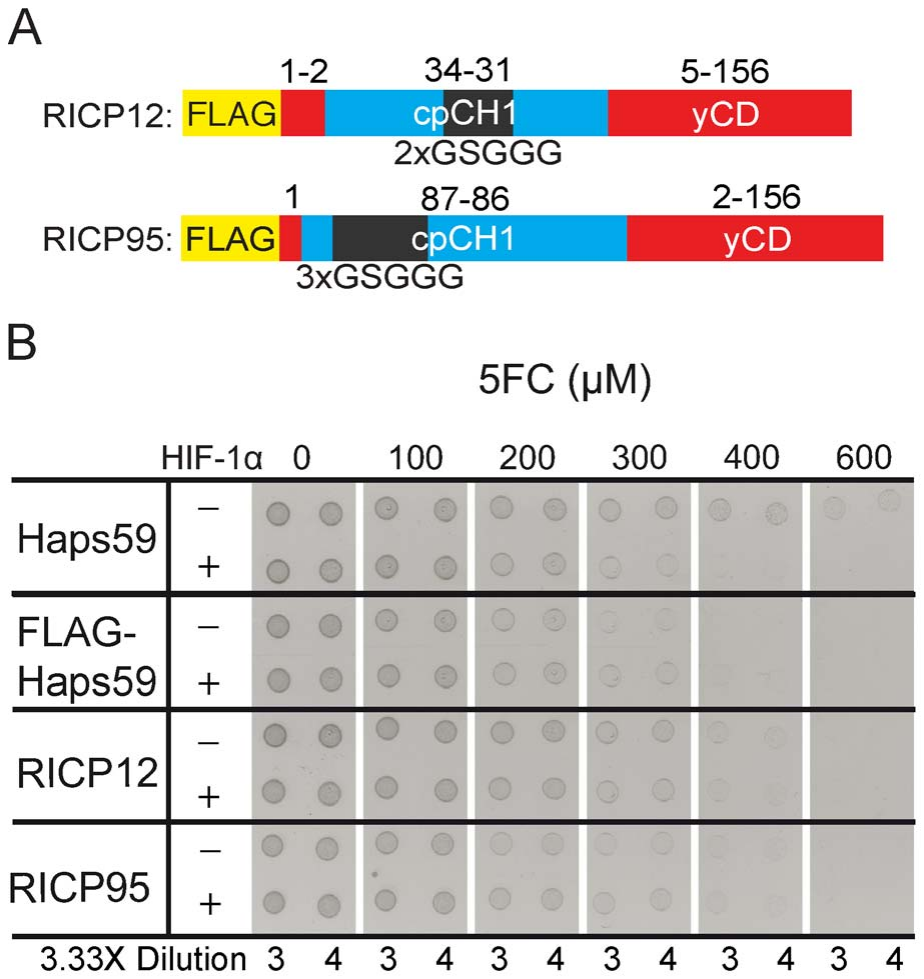
Characterization of RICP12 and RICP95 in *E. coli*. (A) Diagrams of RICP12 and RICP95 sequences identified from the random circular permutation and random insertion library. Numbers above indicate the amino acid residues of the corresponding wild-type proteins. Letters below indicate the 1-letter abbreviation of the amino acid linker sequence used in the circular permutation of the CH1 domain. (B) Dot toxicity assay of RICP12 and RICP95.

For our next library we made a change to Haps59 before library construction in order to help with future characterization in human cells. We appended a FLAG-tag directly to the N-terminus of Haps59 to facilitate characterization of Haps59 in human cell lines, as anti-yCD antibodies suffered from cross reaction with many human proteins in western blots. FLAG-Haps59 and Haps59 exhibited similar HIF-1α-dependent accumulation levels in RKO cells [19], although switching activity of FLAG-Haps59 has not been tested in these cells. Here, we discovered that the addition of the FLAG-tag to Haps59 abolished switching activity in *E. coli* by increasing the 5FC-toxicity in the absence of coexpressed HIF-1α to the same level as in the presence of HIF-1α (Fig 4B). Since the CH1 domain of Haps59 is located very near the N-terminus of the protein, we speculate that the FLAG-tag may stabilize the CH1 domain and the switch.

In an effort to avoid future issues with characterization and increase the potential for being able to purify the resulting switches, we decided to examine if a switch could be constructed starting with FLAG-yCD. We constructed a library of similar design to our previous work [19], in which random circular permutations of the CH1 gene were randomly inserted into the FLAG-yCD gene. However, unlike our previous work, we constructed our libraries using newly developed multiplex PCR methodologies [33], which eliminate out-of-frame members and the majority of non-fusion members, allowing the generation of much more targeted and complete libraries. We created a library of 1.4 million transformants and performed positive and negative selections at different stringencies beginning with 2.5 million cells. Although several members survived both tiers of the selection, the majority of these members contained a CH1 circular permutant inserted in the reverse orientation. None of the few members containing correctly oriented CH1 domains were responsive to HIF-1α (Fig 4B), although the genes contained the CH1 domain inserted near the beginning of yCD like Haps59 (Fig 4A).

### Haps59 is constitutively activated in Flp-In 293 cells

To expedite the translation of Haps, we desired a rapid and reliable method to screen our switches in human cell lines. We had previously characterized Haps59 in RKO and MCF7 cancer cell lines by random stable integration of an encoding vector [19]. This approach requires significant time and resources, and it introduces additional variation in expression level through differences in both the number and location of integrations between clones. This can be avoided using transient expression, however this method is difficult to reproduce consistently and introduces confounding expression dynamics. To avoid these confounding factors we used the Flp-In System (Life Technologies) to generate isogenic single-integration stable cell lines of switches and controls.

In this system, a cell line with a Flp recombinase target (FRT) site stably integrated at a single safe-harbor site in its genome is cotransfected with a Flp recombinase expression plasmid as well as a second plasmid containing the gene to be integrated and a second FRT site. The Flp recombinase mediates recombination between the FRT sites of the vector containing the gene to be integrated and the site stably integrated in the genome. The selectable resistance marker on the vector is initially lacking a start codon, acting as a safeguard against non-specific integration. Only after recombination is the start codon appended and the resistance marker expressed. Additionally this recombination event inactivates the zeocin resistance-LacZ fusion downstream of the FRT site in the genome. Thus one can screen for lack of β-galactosidase activity, to confirm specific integration.

**Figure 5:**
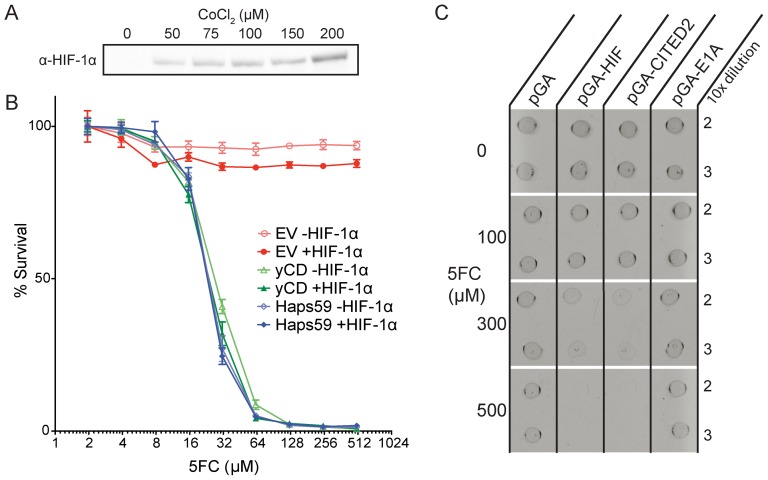
Characterization of the effects of Haps59 expression in Flp-In 293 cells and *E. coli*. (A) Western blot with anti-HIF-1α antibodies showing that the addition of CoCl_2_ causes the accumulation of HIF-1α in Flp-In 293 cells. See Figure S5 for full blot. (B) Toxicity of 5FC to stable isogenic pools of Flp-In 293 cells containing EV, yCD, and Haps59. Cells were grown in the absence (i.e. −HIF-1α) or presence (i.e. + HIF-1α) of 50 µM CoCl_2_, and increasing concentrations of 5FC. Percent survival was calculated by measuring the total DNA of surviving cells in each well and normalizing between wells containing no 5FC (i.e. 100% survival) and wells containing no cells (i.e. 0% survival). Error bars represent the standard deviation across three replicates. (C) Dot toxicity assay indicates that CITED2 but not E1A activates Hap59 in *E. coli*. Equal density dilutions of log phase cultures of strains expressing Haps59 and either the p300 binding fragments of HIF-1α, CITED2, or E1A were plated on increasing concentrations of 5FC.

We integrated Haps59, yCD, and empty vector (EV) controls into Flp-In-293 cells. Three passages (each a 1∶10 dilution) after foci formed on the selection plates, stable pools showed no detectable β-galactosidase activity and were zeocin sensitive, allowing us to assume they are isogenic. These isogenic stable pools were then assayed for ΗIF-1α-dependent 5FC toxicity. ΗIF-1α accumulation was induced with 50 µM CoCl_2_ (Fig 5A) [34].

The negative control EV cell line survived in both the presence and absence of CoCl_2_ up to the highest levels of 5FC tested (512 µM). There was a less than 5% decrease in survival rate due to CoCl_2_ alone. In contrast, cell lines expressing yCD had an EC50 of ∼25 µM regardless of the addition of CoCl_2_. These results are similar to our results with RKO and MCF7 cells [19]. However, Flp-In 293 cells expressing Haps59 exhibited a level of 5FC toxicity nearly equivalent to that of yCD expressing cells in both the presence and absence of CoCl_2_ (Fig 5B). This lack of switching may be partially due to higher expression levels, as 293 cells are commonly known to express high levels of recombinant proteins. In addition, Haps59 may be activated by another binding partner of the promiscuous CH1 domain which is present in 293 cells. The CH1 domain is known to interact with many oncogenes including p53 [35],[36],[37], CITED2 [28],[38],[39],[40], NF-κB-p65 [41],[42], Stat-2 [43],[44], Pit-1 [45],[46],[47], and HNF-4 [48],[49]. One possibility is that activation of Haps59 in Flp-In 293 cells is caused by the interaction between the CH1 domain and the adenovirus E1A protein [50]. The 293 cells were originally created by transformation of sheared adenovirus DNA [51] and have been shown to express E1A [52].

The aberrant activation of Haps59 in Flp-In 293 cells means that these cells have limited use for screening CH1-derived switches identified in *E. coli*. Moreover, the aberrant activation is potentially problematic for two additional reasons. First, it may indicate that domains other than the HIF-1α-CTAD are capable of activating Haps59. While many interactors of CH1 are also oncogenes, Haps59's ability to be activated only in cancer cells is uncertain. Second, the aberrant activation of Haps59 in Flp-In 293 cells indicates that delivery of a *Haps59* gene via viral gene delivery may be problematic, as many viral proteins are known to interact with the CH1 domain [53].

### Haps59 is also activated by CITED2 in *E. coli*

As a first test as to whether domains other than HIF-1α-CTAD that are known to interact with CH1 can activate Haps59, we separately replaced the HIF-1α-CTAD in plasmid pGA-HIF with the CH1-interacting fragments of CITED2 and E1A and tested these constructs' ability to confer increased 5FC toxicity to *E. coli* cells expressing Haps59. CITED2 was chosen as a representative endogenous interactor of CH1 and E1A as a representative viral protein. We found that CITED2 activates Haps59 to a nearly identical extent as HIF-1α, however E1A does not (Fig 5C). We also were unable to coprecipitate E1A with FLAG-Haps59 from lysates of 293-FI cells using anti-FLAG antibodies raising doubt that E1A is responsible for activation of Haps59 in Flp-In 293 cells in the absence of CoCl_2_. In summary, we have found no evidence that the viral E1A protein is responsible for the aberrant activation of Haps59. We have, however, shown that CITED2 is able to activate Haps59 in *E. coli*, supporting the hypothesis that the mis-regulation of some CH1-interacting protein could result in the constitutive activation of Haps59 in Flp-In 293 cells.

## Conclusions and Future Directions

Three second-generation Haps libraries were made spanning unique regions of sequence space. The majority of switches resulting from selections on the three libraries performed equally well or only modestly better than Haps59 in *E. coli*. However, Ehaps22 exhibited a broader therapeutic window in *E. coli* than Haps59, having similar activity in the absence of HIF-1α and significantly increased activity in its presence. The use of random mutagenesis and improved domain insertion and circular permutation techniques were unsuccessful in the identification of improved switches. Additional cycles of random mutagenesis and selection may further improve upon the Ehaps08 and Ehaps22 switches described in this work. While an N-terminal FLAG-tag appears to be a barrier to switch creation, a library including a flexible linker after the FLAG-tag or additional linkers between the CH1 domain and yCD fragments may be more successful. Additionally, it is possible that switches with activity similar to Haps59 in *E. coli* may outperform Haps59 in mammalian cells. However, alternative approaches to the creation of a molecular switch may prove more fruitful.

Screening switches in mammalian cell lines is a highly variable and slow process. In attempt to avoid the variability introduced by random integration as well significantly increase screening throughput, we explored the use of Flp recombinase-mediated isogenic integration [54]. This method of integration is specific, efficient, amenable to high throughput, and produces directly comparable results. Flp-In 293 cells expressing yCD from a stably integrated vector using this method exhibit a similar level of 5FC-dependent toxicity, to previous experiments in RKO and MCF7 cells [19]. However, in Flp-In 293 cells expressing Haps59, this toxicity is independent of HIF-1α. This may be due to the promiscuous binding activity of the CH1 domain, the significant genetic abnormality of 293 cells, and/or possible expression level differences between Flp-In 293 cells and RKO/MCF7 cells. In future work the Flp-In system will be used to integrate the new switches identified here into RKO and MCF7 cells in order to evaluate their performance in human cells.

Our experiments in Flp-In 293 cells have raised the potential for off-target activation of Haps switches. To alleviate the related concerns, we propose future *in vivo* murine experiments utilizing a panel of systemic delivery vehicles to characterize the specificity of these therapeutic switches. Off-target activity can be traced using [^19^F]-labeled 5FC [55],[56]. Should significant off-target activity be found, aberrant activators in normal tissue may then be co-precipitated with the switch and identified by protein mass-spectrometry. Further directed evolution can then be performed to abolish this off-target activity while retaining HIF-1α activation. Alternatively, off-target activity might be avoided by placing the switch genes under the control of cancer specific promoters (e.g. transcriptional regulation).

## Materials and Methods

### Strains, Plasmids, and Reagents

All cloning was performed in DH5α (Life Technologies). GIA39 *(thr-1 leuB6(Am) fhuA21 codA1 lacY1 tsx-95 glnV44(AS) λ^−^ dadX3 pyrF101 his-108 argG6 ilvA634 thiE1 deoC1 glt-15)* (Coli Genetic Stock Center) was used for all selections and screening. The genes encoding Haps59 and yCD were cloned into pSkunk (p15a origin, f1 origin, AadA streptomycin/spectinomycin resistance, and tac promoter) using primers encoding a 5′ NdeI site and a 3′ SpeI site. Plasmids pGA and pGA-HIF are ColE1 and *BLA* containing plasmids which express GST or GST-HIF-CTAD from the arabinose promoter [19]. pGA-CITED2 and pGA-E1A were made by initially synthesizing the coding sequence for amino acids 220–269 of CITED2 using overlapping oligonucleotides [57] and PCR amplification of the coding sequence of amino acids 1–77 of Adenovirus 5 E1A from genomic DNA of 293 cells. Homologous regions of the 3′ portion of GST and downstream flanking vector were added to the CITED2 and E1A fragments by PCR. Primers for adding these homologous flanking regions were designed using j5 [58]. These fragments were incorporated into pGA backbone using circular polymerase extension cloning [59]. Any unspecified cloning was performed using CPEC [59] with primers designed in j5 [58]. All enzymes were obtained from NEB and all chemicals were obtained from Thermo-Fisher or Sigma unless specified.

### Error-prone PCR

Initially a PCR was performed using Phusion HF DNA polymerase with primers flanking genes encoding Haps59 to generate a 902 bp template for the error-prone reaction. This template was purified using a Zymo DNA clean and concentrate (DCC) 5 column (Zymo Research) and error prone PCR was performed as previously described [60] to achieve an average of approximately 3 nucleotide substitutions per 1000 bases. Library size calculations were performed according to Bosley and Ostermeier [32]. The resulting PCR product was purified using a Zymo DCC column and digested with NcoI and SpeI. The digested fragment was electrophoresed on an agarose gel and purified using Qiagen Gel Purification columns (Qiagen). This purified fragment was ligated into similarly digested pSkunk backbone using T4 DNA polymerase overnight at 16°C. DH5α cells were electroporated with the resulting library and plated on a 24.5×24.5 cm bioassay dish of LB agar containing 50 µg/mL streptomycin and 0.2% glucose. After overnight growth, this naïve library was recovered from the plate and plasmid DNA was isolated by miniprep (Qiagen) for transformation into selection strains, as described below.

### Linker Library Creation

Oligonucleotides encoding 0, 1, 2, and 3 NNK degenerate codons and annealing to the fusion points between the N-terminal portion of yCD and the CH1 domain and the CH1 domain and the C-terminal portion of yCD were synthesized by IDT. Each of the 16 N- and C-terminal linker pairs of oligos was used to perform Kunkel Mutagenesis [61]. For this technique, single-stranded uracil-containing DNA was purified from phage, which was produced by CJ236 (*dut^−^ ung^−^* F1^+^, Coli Genetic Stock Center) containing pSkunk-Hap59 grown in the presence of R408 helper phage (Promega). One µg of this DNA was used as a template for second strand synthesis primed by the phosphorylated random linker encoding oligos in a reaction containing T4 polymerase and T4 ligase. The resulting products were electroporated into DH5α cells, which degrade the uracil-containing strand and sythesize a new complement strand now containing the mutations. Cells were plated and library DNA was prepared as above.

### Random insertion and circular permutation library

As a template for multiplex inverse PCR [33], which generates random insertion sites in yCD, DNA encoding a FLAG tag was appended to the 5′ end of the gene encoding yCD and cloned into pSkunk. As templates for circular permutation of the CH1 domain, two copies of the CH1 domain connected by a 10mer (2×GSGGG) and 15mer (3×GSGGG) linker were cloned into pCDNA3.1 (Life Technologies). Abutting primers were designed using a Matlab program developed in-house (available upon request) and were obtained from IDT. Multiplex PCR was performed as described previously [33], creating direct insertions/permutation as well as duplication and deletions of 2 amino acids at each position within yCD and CH1. PCR success and quality was verified by agarose gel electrophoresis and reactions were pooled, purified, and concentrated by phenol/chloroform extraction followed by ethanol precipitation. Products of the correct sizes were isolated by agarose gel electrophoresis extraction using PureLink Gel Purification columns (Life Technologies). The resulting DNA was further purified by phenol/chloroform extraction and ethanol precipitation. The pool of circularly permuted CH1 domains were phosphorylated using NEB Quick Blunting Kit, per the manufacturers directions. This reaction was used directly in a blunt-ended ligation reaction with the pool of randomly opened pSkunk-FLAG-yCD vectors, per the previously described protocol [33]. The naïve library DNA was transformed, amplified, and prepared as above.

### Selections

Following the isolation of naïve library DNA, 25 ng was transformed into GIA39 cells for negative selections. Transformants were plated on LB agar containing 0.2% glucose and 50 µg/mL streptomycin. After overnight incubation the resulting colonies were recovered from the plate with minimal sweep media (1×M9 salts, 2% glucose, 15% glycerol) and the collected cells were centrifuged for 10 minutes at 3000 *g*. Supernatant was discarded and library members were resuspended in 1 cell pellet volume of fresh minimal sweep media. Small aliquots were then stored at −80°C. A single aliquot was thawed and several dilutions were plated on unselective solid media (1× yeast nitrogen base, 1× yeast dropout media without uracil, 2% select agar, 5 µg/mL uracil, 2% glucose, and 50 µg/mL streptomycin, 1 mM IPTG) to determine the concentration of viable cells. Based on this concentration, 600,000 cells were plated per 24.5 cm square bioassay dish in each selection, unless this was greater than 10 times the library size, in which case 10 times the maximum theoretical diversity of the library was plated. Dilutions were plated on 10 cm petri dishes of selective and unselective media to confirm the number of cells plated and calculate the number of surviving members on the bioassay dish.

For negative selections, library members were plated on unselective media supplemented with 969 µM 5-fluorocytosine (or between 500 and 969 µM for the random insertion and circular permutation library). After 18–24 hours of growth at 37°C, surviving members were recovered from the plate in minimal sweep media by sweeping, centrifuged at 3000xg for 10 minutes, resuspended in 1 cell pellet volume of fresh minimal sweep media, aliquoted, and stored at −80°C. If greater than 30% of the plated members survived the selection, the selection was repeated. If not, library plasmid DNA was prepped from an aliquot of the selected members and GIA39 cells harboring pGA-HIF (GIA39-HIF) were electroporated with 25 ng of this DNA and plated on LB agar containing 0.2% (w/v) glucose, 100 µg/mL carbenicillin, and 50 µg/mL streptomycin. Selected library members were collected as described above.

Negatively selected libraries in GIA39-HIF were plated on positive selection plates of dropout media without uracil (1× yeast nitrogen base, 1× yeast dropout media without uracil, 2% select agar, 2% glucose, 50 µg/mL streptomycin, 100 µg/mL carbenicillin, 1 mM IPTG, 0.05% (w/v) L-arabinose) supplemented with 625 ng/mL cytosine (or between 500 ng/mL and 5 µg/mL for the random insertion and circular permutation library). Selective plates were incubated at 37°C for 36 hours prior to picking the largest colonies for screening.

### Screening in *E. coli* and dot toxicity assays

Colonies isolated from positive selection plates were struck out on unselective plates with and without L-arabinose and supplemented with 300 µM 5FC (as well as an LB plate for further propagation) as an initial assessment of switching. In cases where a large number of colonies survived the final tier of selection, these colonies were inoculated in 1 mL unselective minimal media in a 96-well block, struck out on LB plates, and then incubated overnight at 37°C with constant shaking. In the morning the liquid cultures were diluted 1/100 into fresh media and incubated for an additional 4–6 hours, before diluting and spotting (as described below) on unselective plates with and without L-arabinose and supplemented with 300 µM 5FC. Growth on plates with and without L-arabinose was then compared to assess switching.

Colonies from the LB plates of members with the largest ratio of growth in the absence versus presence of L-arabinose were grown overnight in LB media containing 50 µg/mL streptomycin. Plasmid DNA was isolated by miniprep and sequenced. These members were then subcloned into fresh pSkunk vector and confirmed by Sanger sequencing. Chemically competent [62] GIA39 containing pGA or pGA-HIF were transformed with the subcloned switches. Fresh colonies of these strains were inoculated into unselective liquid dropout media (1× yeast nitrogen base, 1× yeast dropout media without uracil, 10 µg/mL uracil, 2% glucose, 100 µg/mL ampicillin, and 50 µg/mL streptomycin) and incubated overnight with constant shaking at 37°C. Overnight cultures were diluted 1/100 into fresh unselective liquid media and grown for 4–6 hours until OD_600_ was greater than 0.3. Cultures were diluted to 0.3 OD_600_ and serial dilutions were made such that the OD_600_ of the final dilution was less than 0.0003. One µL of each dilution was spotted on unselective solid media supplemented with 0.05% (w/v) L-arabinose, 100 µg/mL ampicillin, and containing increasing concentrations of 5-FC in omnitrays (Nunc) and grown overnight at 37°C. Plates were imaged under white light using a Biorad Universal Doc II and Quantity One software. Members exhibiting a high ratio of growth in GIA39-pGA relative to GIA39-HIF cells were chosen for further study. Similar assays were performed using pSKunk-Haps59 with pGA, pGA-HIF, pGA-CITED2 or pGA-E1A in GIA39 cells to test the ability of CITED2 and E1A to activate Haps59.

For dot toxicity assays, plate images (Fig S1, S2, S3 and S4) were inverted and cropped using ImageJ [63]. Using the ImageJ Dot Blot Analyzer macro [64], background was subtracted from the images using the paraboloid 2D rolling ball method with a diameter of 25. A grid was fit to the dots including an extra column where no culture was spotted, as a measurement of no growth, and spot intensities were quantified. Spots with intensities more than 4 standard deviations larger than the no-growth-control average for each plate were considered the last surviving dilutions.

### Isogenic stable pools

Flp-In 293 cells (catalog number R750-07), pCDNA5/FRT/TO, and pOG44 were obtained from Life Technologies. Flp-In 293 cells were maintained in DMEM high-glucose w/L-glutamine supplemented with an additional 2 mM L-glutamine (Gibco) and 100 µg/mL zeocin (Life Technologies). Haps59 and yCD were cloned into pCDNA5/FRT/TO, and DNA for transfections was prepped from overnight cultures of *E. coli* using PureYield Miniprep kit (Promega). Α total of 2.7 µg of pOG44 and 300 ng of pCDNA5/FRT/TO-EV, -yCD, and -Haps59 were transfected into 100,000 Flp-In 293 cells, plated eighteen hours prior in a single well of a 6-well plate using the CalPhos Mammalian Transfection Kit (Clontech) following the manufacturers directions. Twelve hours after transfection the cells were washed with PBS and fresh media was added. Thirty-six hours after transfection the cells were split 1 to 5 into a fresh 6-well plate of media containing 150 µg/mL hygromycin-B, instead of zeocin. Media was changed every 3–4 days until foci formed, in about 2 weeks. These stable pools were passaged 3 times, at a 1/10 dilution into 200 µg/mL hygromycin-B, before being used in toxicity assays.

### HIF-1α Western Blot

Flp-In 293 cells were grown to 75% confluence in 10 cm dishes and media was changed to contain increasing concentrations of CoCl_2_ and incubated for 4 hours. Lysis and western blotting was performed as previously described [19].

### Mammalian cell toxicity assays

The inner 60 wells of a 96 well plate were seeded with 1000 cells per well in 100 µL of media containing 200 µg/mL hygromycin-B. The next day 50 µL of media was added to achieve the desired final concentrations of 5FC and 50 µM CoCl_2_ (in +HIF samples). Cells were incubated for 4 days prior to washing twice with PBS and freezing at −80°C to lyse. Total DNA was measured by adding 200 µL of 0.075% SYBR green (Life Technologies) in sterile filtered deionized water to each well, pipetting up and down 7 times, incubating at 37°C for 4–6 hours, and reading the fluorescent emission at 535 nm after excitation at 485 nm with a 515 nm cutoff filter.

## Supporting Information

Figure S1 **Ehaps22 and Ehaps08 dot toxicity assay replicate 1.** Serial dilutions of equal density log phase cultures containing either pGA (−HIF-1α, i.e. not expressing HIF-1α) or pGA-HIF (+HIF-1α, i.e. expressing HIF-1α) were spotted on minimal media plates containing increasing concentrations of 5FC. Plate images here are background subtracted.
(TIF)

Figure S2 **Ehaps22 and Ehaps08 dot toxicity assay replicate 2.**
(TIF)

Figure S3 **Ehaps22 and Ehaps08 dot toxicity assay replicate 3.**
(TIF)

Figure S4 **Ehaps22 and Ehaps08 dot toxicity assay replicate 4.**
(TIF)

Figure S5 **Western blot with anti-HIF-1α antibodies showing that the addition of CoCl_2_ causes the accumulation of HIF-1α in Flp-In 293 cells.** Bands near 100 kDa indicate full length HIF-1α (94 kDa). We hypothesize that bands near 120 kDa indicate ubiquitinated HIF-1α (HIF-1α-Ub). Unidentified bands between 50 and 60 kDa were also detected.
(TIF)
