# Supplementary figures and images for: Advancements in the Development of HIF-1α-Activated Protein Switches for Use in Enzyme Prodrug Therapy
Source: PLoS One. 2014 Nov 26;9(11):e114032. doi: 10.1371/journal.pone.0114032 (PMC4245239; doi:10.1371/journal.pone.0114032)

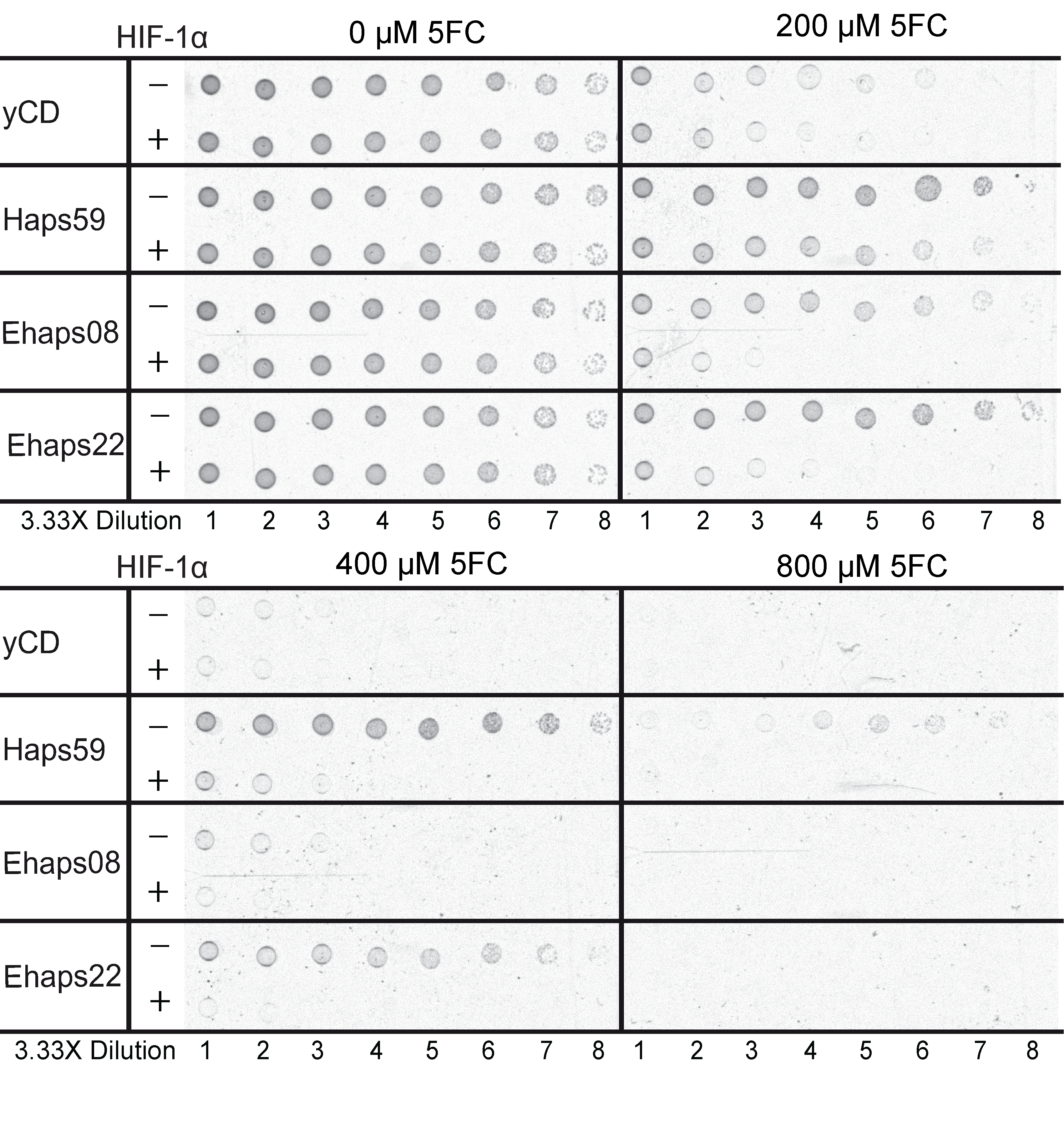

Supplement: Figure S1 — Ehaps22 and Ehaps08 dot toxicity assay replicate 1. Serial dilutions of equal density log phase cultures containing either pGA (−HIF-1α, i.e. not expressing HIF-1α) or pGA-HIF (+HIF-1α, i.e. expressing HIF-1α) were spotted on minimal media plates containing increasing concentrations of 5FC. Plate images here are background subtracted. (TIF) [file pone.0114032.s001.tif]

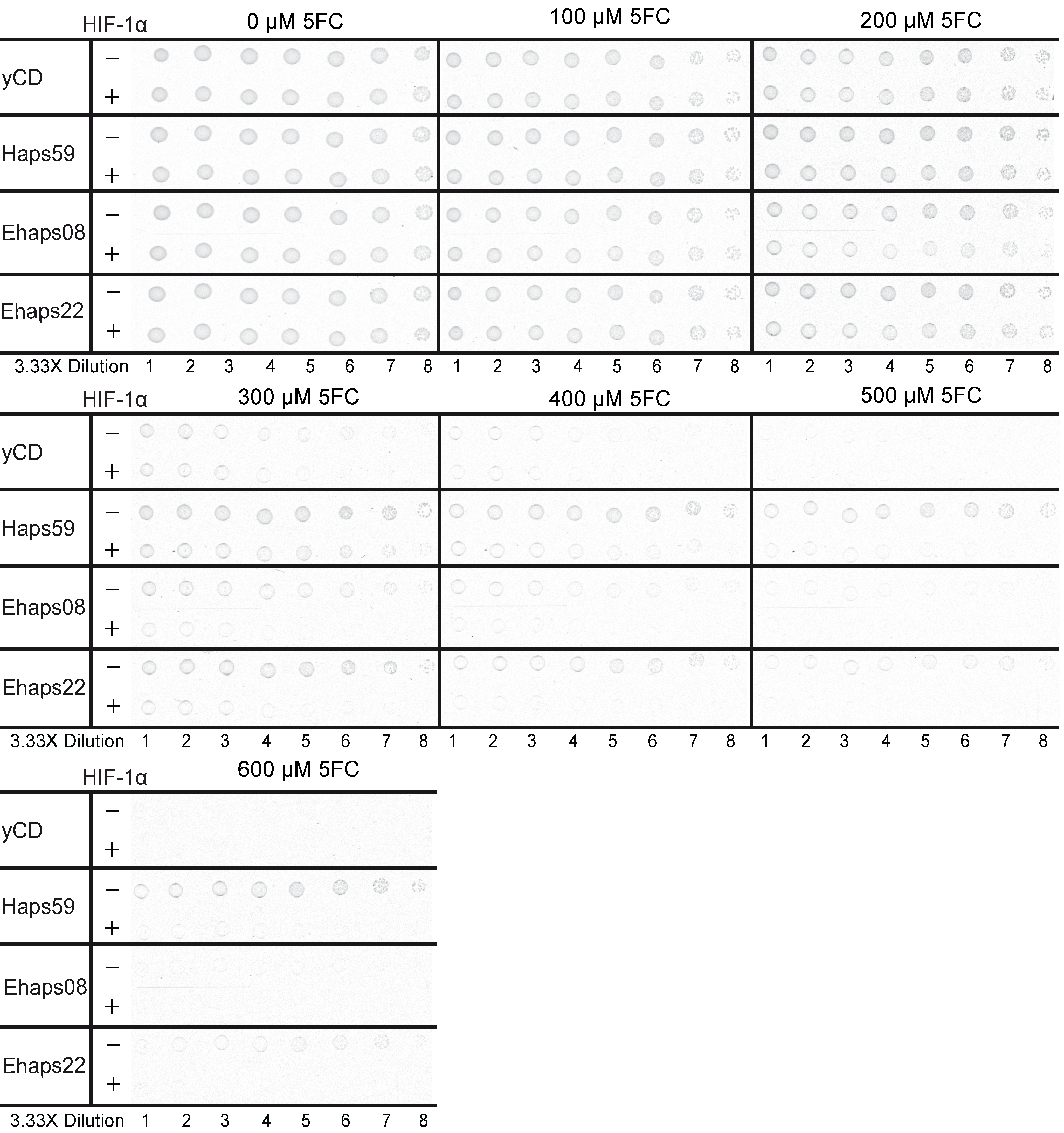

Supplement: Figure S2 — Ehaps22 and Ehaps08 dot toxicity assay replicate 2. (TIF) [file pone.0114032.s002.tif]

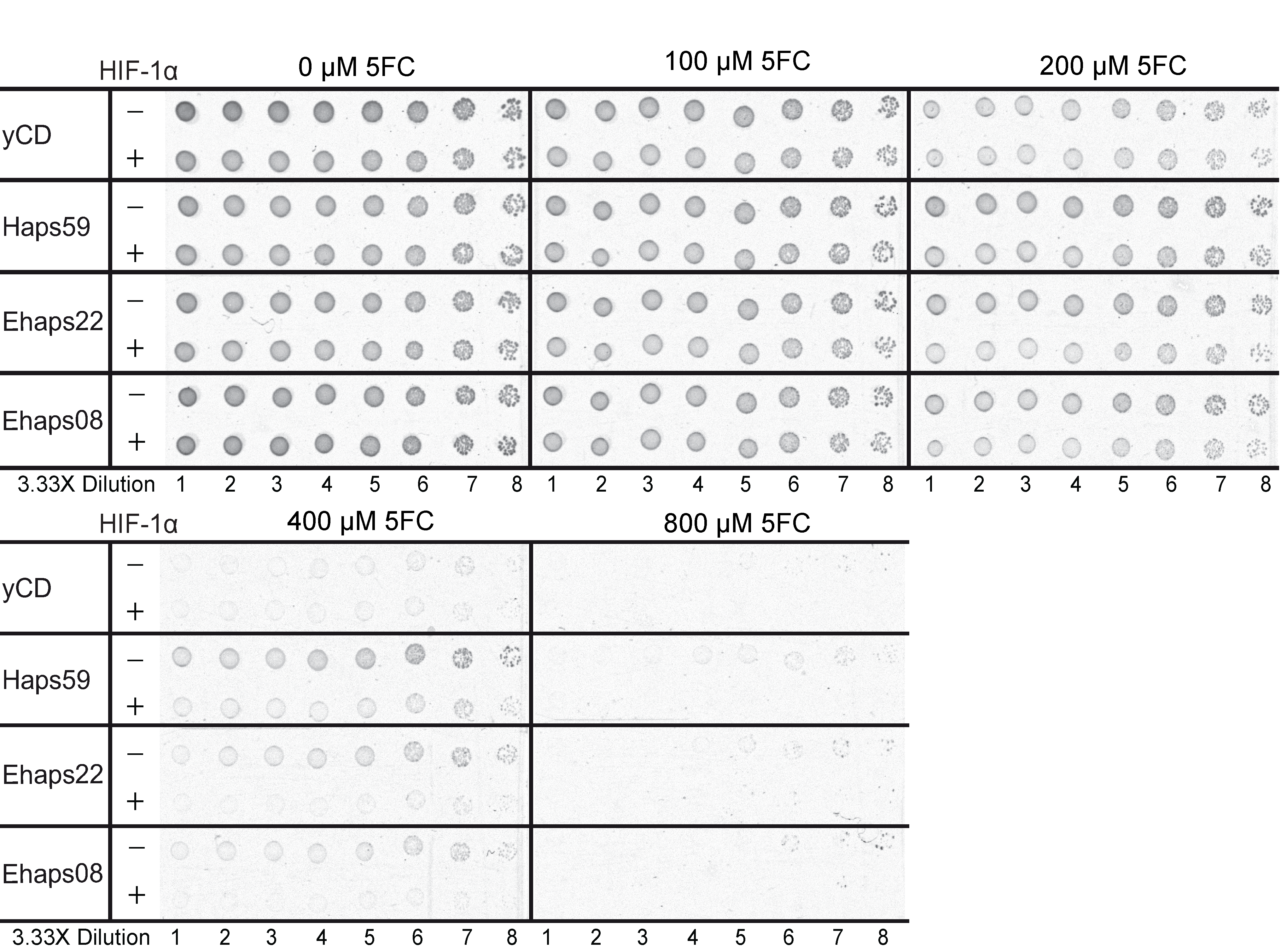

Supplement: Figure S3 — Ehaps22 and Ehaps08 dot toxicity assay replicate 3. (TIF) [file pone.0114032.s003.tif]

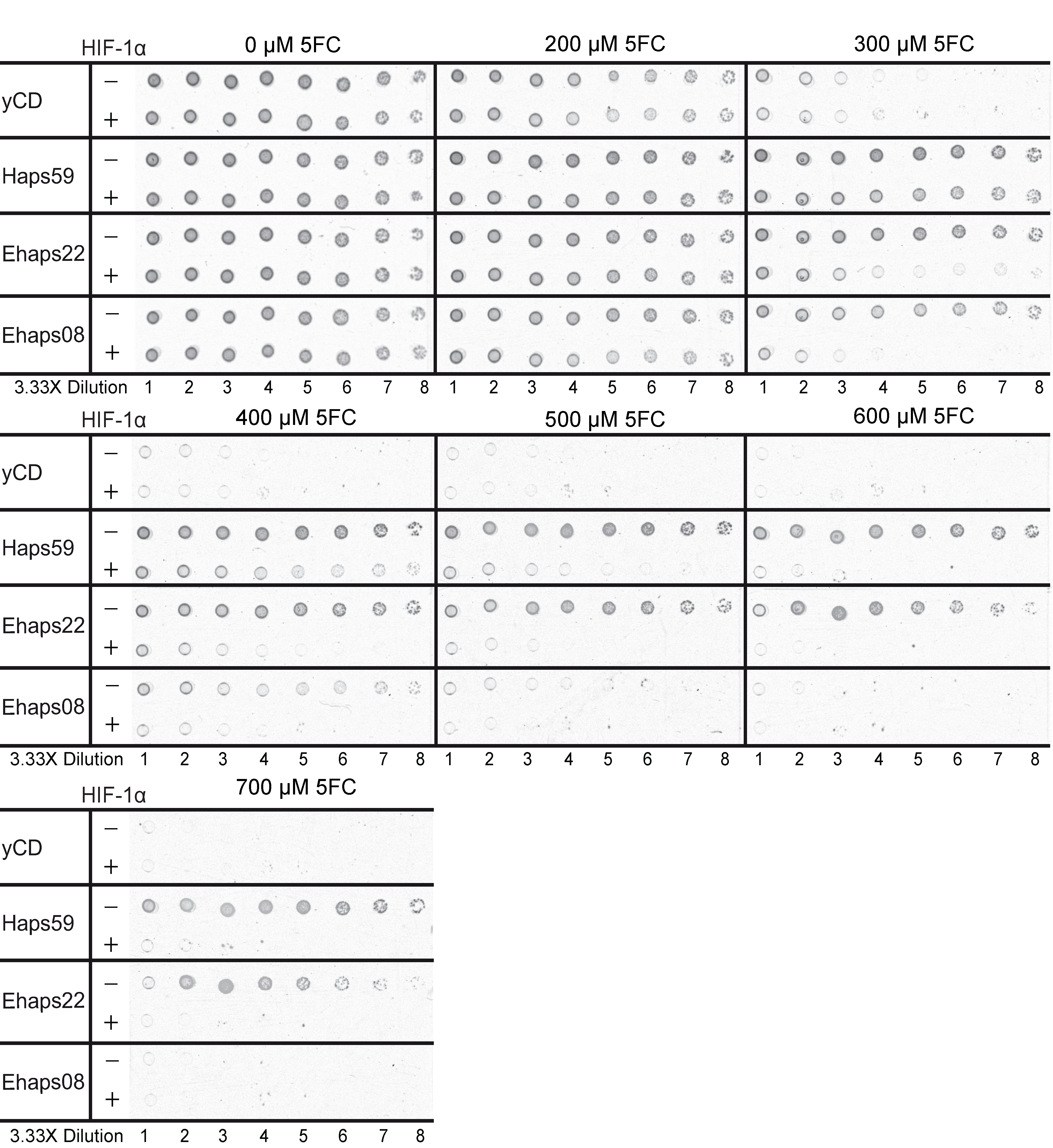

Supplement: Figure S4 — Ehaps22 and Ehaps08 dot toxicity assay replicate 4. (TIF) [file pone.0114032.s004.tif]

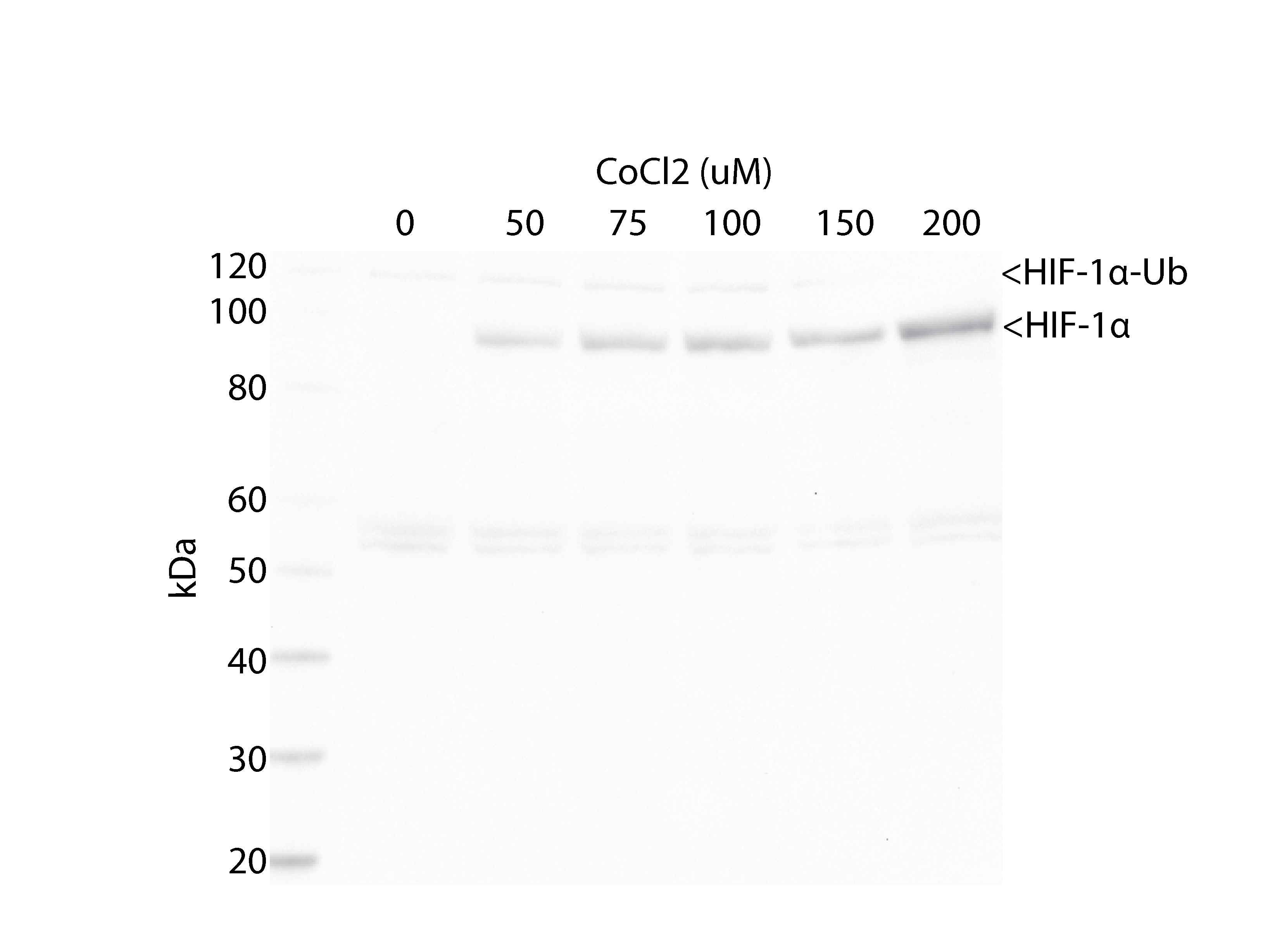

Supplement: Figure S5 — Western blot with anti-HIF-1α antibodies showing that the addition of CoCl2 causes the accumulation of HIF-1α in Flp-In 293 cells. Bands near 100 kDa indicate full length HIF-1α (94 kDa). We hypothesize that bands near 120 kDa indicate ubiquitinated HIF-1α (HIF-1α-Ub). Unidentified bands between 50 and 60 kDa were also detected. (TIF) [file pone.0114032.s005.tif]
